# Microsphere-Based Hierarchically Juxtapositioned Biphasic Scaffolds Prepared from Poly(Lactic-*co*-Glycolic Acid) and Nanohydroxyapatite for Osteochondral Tissue Engineering

**DOI:** 10.3390/polym8120429

**Published:** 2016-12-10

**Authors:** K. T. Shalumon, Chialin Sheu, Yi Teng Fong, Han-Tsung Liao, Jyh-Ping Chen

**Affiliations:** 1 Department of Chemical and Materials Engineering, Chang Gung University, Kwei-San, Taoyuan 33302, Taiwan; chialinsheu@gmail.com; 2 Department of Plastic and Reconstructive Surgery and Craniofacial Research Center, Chang Gung Memorial Hospital, Kwei-San, Taoyuan 33305, Taiwan; evausatw@gmail.com (Y.T.F.); lia01211@gmail.com (H.-T.L.); 3 Graduate Institute of Health Industry and Technology, Research Center for Industry of Human Ecology, Chang Gung University of Science and Technology, Kwei-San, Taoyuan 33302, Taiwan; 4 Department of Materials Engineering, Ming Chi University of Technology, Tai-Shan, New Taipei City 24301, Taiwan

**Keywords:** microspheres, poly(lactide-*co*-glycolide), nanohydroxyapatite, scaffold, tissue engineering, osteochondral, biphasic

## Abstract

This study aims to prepare biphasic osteochondral scaffolds based on seamless joining of sintered polymer and polymer/ceramic microspheres for co-culture of chondrocytes and bone marrow stem cells (BMSCs). Poly(lactide-*co*-glycolide) (PLGA) microspheres and 10% nanohydroxyapatite (nHAP)-incorporated PLGA (PGA/nHAP) microspheres were prepared through the oil-in-water precipitation method. Virgin (V) and composite (C) scaffolds were prepared from 250–500 µm PLGA and PLGA/nHAP microspheres, respectively, while osteochondral (OC) scaffolds were fabricated through the combination of V and C scaffolds. Physico-chemical properties of scaffolds were characterized through microscopic-spectroscopic evaluations. The effect of nHAP in scaffolds was investigated through thermogravimetric analysis and mechanical testing, while surface hydrophobicity was tested through contact angle measurements. Rabbit chondrocytes and BMSCs were used for cell culture, and cell morphology and proliferation were determined from SEM and DNA assays. Alizarin red and Alcian blue stains were used to identify the in vitro bone and cartilage tissue-specific regeneration, while cetylpyridinium chloride was used to quantitatively estimate calcium in mineralized bone. For co-culture in OC scaffolds, BMSCs were first seeded in the bone part of the scaffold and cultured in osteogenic medium, followed by seeding chondrocytes in the cartilage part, and cultured in chondrocyte medium. High cell viability was confirmed from the Live/Dead assays. Actin cytoskeleton organization obtained by DAPI-phalloidin staining revealed proper organization of chondrocytes and BMSCs in OC scaffolds. Immunofluorescent staining of bone (type I collagen and osteocalcin (OCN)) and cartilage marker proteins (type II collagen (COL II)) confirmed cellular behavior of osteoblasts and chondrocytes in vitro. Using an ectopic osteochondral defect model by subcutaneous implantation of co-cultured OC scaffolds in nude mice confirmed cell proliferation and tissue development from gross view and SEM observation. IF staining of OCN and COL II in the bone and cartilage parts of OC scaffolds and tissue-specific histological analysis exhibited a time-dependent tissue re-modeling and confirmed the potential application of the biphasic scaffold in osteochondral tissue engineering.

## 1. Introduction

Osteochondral defects are major tissue defects caused by traumatic injury or disease, due to the damage of both articular cartilage and underlying subchondral bone [1]. Osteochondral grafting, prosthetic joint replacement, and chondroplasty are the available methods for osteochondral repair. However, donor site morbidity, abrasion, surface loosening of implants, and risk of infection put osteochondral tissue engineering as a high demand task [2]. In osteochondral tissue engineering, it provides a favorable alternative therapy to engineer a scaffold with the same structural and mechanical properties as a native cartilage–bone plug [3,4,5]. A suitably designed scaffold architecture is important for the individual formation of bone [6,7,8], cartilage [9,10,11] or both, in osteochondral defects, with the combinatory approach being of greatest interest owing to the difficulty in achieving the goal [12,13]. The fabrication of such biphasic scaffolds, which can control the formation of a composite bone and cartilage architecture, remains a significant challenge.

Osteochondral defect repair or regeneration requires three components; bone, cartilage, and bone–cartilage interface. Though several studies have undertaken for the better understanding of bone and cartilage tissue engineering over the years, still, osteochondral tissue repair is unlike these two separate areas. The true challenge in osteochondral tissue repair lies in the effectiveness of osteochondral interface formation, its mechanical strength, structure, and biology. Considering the structure–morphology relations, a bi-phasic or bi-layer scaffold design is more appropriate for facing such challenges. Each layer of the scaffold should be engineered to exhibit tissue-specific biophysical conditions and environments suitable to support the regeneration of two tissue types: articular cartilage and subchondral bone. Only few reports are available related to integration of bi-layered scaffolds for osteochondral tissue engineering using natural or synthetic polymers [14,15,16,17,18]. Bi-layered scaffolds can be made in two ways: separately engineering the bone and cartilage tissues and combining them together or engineering both tissue types in an integrated scaffold. Compared to the former one, integrated bi-layered scaffolds provide a gradual transition of bone and the cartilage within a single scaffold, thus avoiding the procedure to joint both parts before implantation. Various methods have been reported for the fabrication of osteochondral scaffolds in which a three-dimensional (3D) printing/salt leaching technique for the production of cartilage and bone regions using poly(lactic-*co*-glycolic acid)/poly(lactic acid) (PLGA/PLA) and PLGA/tricalcium phosphate was reported [19]. A two-stage crosslinking procedure composing oligo(poly(ethylene glycol) fumarate) (OPF) and OPF/gelatin microparticles in an integrated bi-layered scaffold was reported [20]. Chen et al. reported the development of a bi-layered scaffold through a combined solvent casting, salt leaching, and freeze drying method for making bi-layered scaffolds of collagen and PLGA/collagen for cartilage and bone layers, respectively [21]. However, most of the reports do not meet the requirements, such as mechanical strength, porosity, moldability, and pore interconnectivity [22], which are essential in developing the best osteochondral scaffold for tissue engineering.

Numerous biomaterials such as proteins or carbohydrate-based polymers, including collagen, hyaluronan, and chitosan, and synthetic materials such as bioactive ceramics and synthetic polymers are being investigated for interface tissue engineering [23,24,25,26]. Though natural materials are superior in their biological interaction with host tissues, their relative mechanical inferiority and instability compared with native cartilage makes them inappropriate for clinical applications. At the same time, having relatively low biocompatibility with natural materials, synthetic materials have high controllability in their chemical, mechanical, and structural properties and thereby could satisfy the increasing clinical demand. Considering their excellent osteoconductive/osteoinductive capabilities [17,27,28], synthetic bioactive ceramics such as nanohydroxyapatite (nHAP), tricalcium phosphate, silicate bioactive glass, *etc*. could be used in the bone part of the bi-layered scaffold for osteochondral tissue engineering.

The cell type used for osteochondral repair has a great influence on its overall outcome. A controlled environment is required to prevent cell differentiation into another phenotype. That is, to prevent chondrocytes from differentiating into fibroblasts or to arrest stem cell differentiation at the chondrocyte stage without further proceeding to ossification [29]. Similarly, cells in a bone compartment should be able to maintain their own phenotype, without getting disturbed by surroundings or related factors. Hence, incorporation of growth factors was put forward, to enhance the cell performance in vivo. The function of growth factors is to establish an inducing environment for cells to differentiate into a specific phenotype. Either single growth factor or a combination of growth factors was used in osteochondral tissue development. Various reports are available on the use of single or multiple growth factors in osteochondral regeneration [30,31,32]. However, growth factors have not been validated. Besides, due to the inability to control the release of growth factors precisely to designated parts in osteochondral scaffolds, both cell types will experience inductive effects from both growth factors and will eventually regenerate a single cell phenotype. It should be noted that incorporating growth factors or platelet-rich plasma (PRP) in the osteochondral scaffold could support angiogenesis/vascularization [33].

In this work, we are approaching with a microsphere-based PLGA-nHAP/PLGA integrated scaffold with hierarchically designed PLGA/nHAP microspheres in the bone part while with PLGA microspheres in the cartilage part. The osteo-conductive nHAP was incorporated in situ to achieve uniform distribution within the bone part of the scaffold to induce differentiation. We postulate that since nHAP could induce osteogenic differentiation of bone marrow stem cells (BMSCs) that are in direct physical contact with the bone part of the scaffold, a stem cell-based approach could be used here to first create the bone part of an osteochondral zone. Pre-differentiation of BMSCs to the osteogenic lineage was achieved through the combined effect of osteo-conductive PLGA/nHAP in osteogenic medium (OM) for successful creation of the new bone tissue. Subsequently, chondrocytes were seeded in the cartilage part, maintained in the chondrocyte medium and evaluated for osteochondral tissue regeneration in vitro and in vivo.

## 2. Materials and Methods

### 2.1. Materials

PLGA with a lactide-to-glycolide ratio of 85:15 (intrinsic viscosity 0.96 dL/g) was purchased from Green Chemical Inc., Taipei, Taiwan, while calcium hydrogen phosphate (Ca_2_HPO_4_·2H_2_O) was purchased from Showa Co., Gyoda, Japan. Calcium carbonate (CaCO_3_), sodium hydroxide (NaOH), cetylpyridinium chloride and poly(vinyl alcohol) were obtained from Sigma-Aldrich (St. Louis, MO, USA). Dichloromethane (CH_2_Cl_2_) was procured from Alfa Aesar, Ward Hill, MA, USA, while Dulbecco’s Modified Eagle’s Medium (DMEM) and fetal bovine serum (FBS) were purchased from Invitrogen (Carlsbad, CA, USA) and Thermo Fisher Scientific (Waltham, MA, USA), respectively. Dulbecco’s Modified Eagle’s Medium/Nutrient Mixture F-12 (DMEM/F-12) was purchased from Sigma-Aldrich (St. Louis, MO, USA). All chemicals were used as received without further purification.

### 2.2. Preparation of Nanohydroxyapatite (nHAP)

The chemical precipitation method using Ca_2_HPO_4_·2H_2_O and CaCO_3_ was used to prepare nHAP particles, as reported in our previous studies [34]. Briefly, 0.86 g of CaHPO_4_·2H_2_O and 0.335 g of CaCO_3_ were gently mixed in 2.5 M NaOH solution at 75 °C for 1 h, followed by termination of the reaction by keeping the mixture in an ice bath. The resultant solution was centrifuged, multiple washed with double distilled water, and dried at 70 °C for 24 h to obtain nHAP. 

### 2.3. Preparation of PLGA and PLGA/nHAP Microspheres

**Figure 1:**
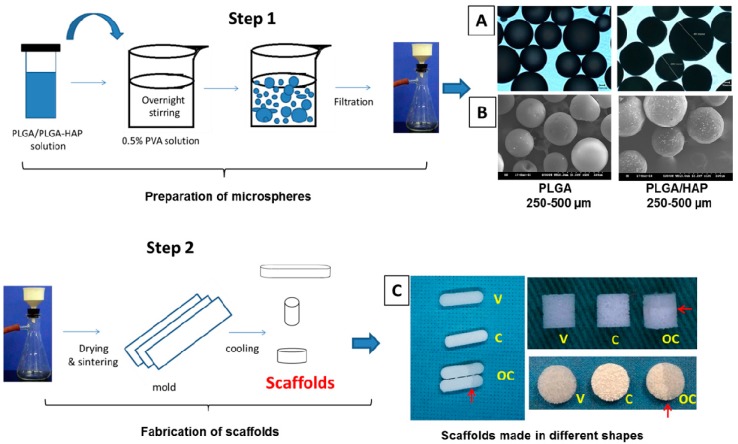
Schematic representation of preparation of PLGA and PLGA/nHAP (Poly(lactide-*co*-glycolide)/nanohydroxyapatite) microspheres by the oil-in-water emulsion/solvent evaporation method (Step 1) and the corresponding light microscopic (**A**) and SEM images (**B**) of microspheres in the range of 250–500 µm. Step 2 represents the sintering of microspheres to make virgin (V), composite (**C**) and osteochondral (OC) scaffolds of various shapes with the gross views shown in (**C**). Red arrows in (**C**) point out the interface regions in the curve-edged rectangular, disc, and cylindrical-shaped OC scaffolds with distinguishable separating zones for cartilage and bone parts, respectively.

Both PLGA and PLGA/nHAP microspheres were prepared by the oil-in-water emulsion/solvent evaporation method, as reported by Yang et al. [35]. For PLGA microspheres, 4.2 g PLGA was dissolved in 30 mL dichloromethane to get 14% PLGA solution, followed by slowly pouring into 1.6 L polyvinyl alcohol (PVA) solution (0.5%) and stirred overnight at 360 rpm. For PLGA/nHAP microspheres with 15% of nHAP, 4.2 g PLGA was mixed in 30 mL dichloromethane with 630 mg nHAP and allowed to undergo microsphere formation through overnight stirring in 0.5% PVA solution. Formed microspheres were washed with distilled water, dried under vacuum, and sorted using metallic sieves (Figure 1, step 1).

### 2.4. Fabrication of Scaffolds

Selected microspheres with size ranging from 250 to 500 μm were sintered in a pre-designed stainless steel mold at 85 °C for 90 min to produce three types of scaffolds: virgin (V) scaffolds using PLGA microspheres, composite (C) scaffolds using PLGA/nHAP microspheres, and osteochondral (OC) scaffolds with hierarchically juxtapositioned V and C scaffolds in a single biphasic scaffold using PLGA and PLGA/nHAP microspheres. The scaffolds could be made into various shapes such as cylinders (4 mm diameter × 4 mm height), discs (6 mm diameter × 2 mm height), and curve-edged rectangles (18 mm length × 4 mm width × 2 mm height) (Figure 1, Step 2) to be used in various experiments.

### 2.5. Characterization of Scaffolds

The size and morphology of the prepared nHAP was determined using a transmission electron microscope (TEM) (JEM-2000EXII, JEOL, Tokyo, Japan) at 75 kV, whereas the crystallographic phases of nHAP were examined through an X-ray diffractometer (XRD) (D5005, Siemens AG, Munich, Germany) with a CuKa source (wavelength = 1.54056 Å), a goniometric plate, and a quartz monochromater having a scanning speed of 2°/min from 20° to 60°. A Horiba FT-730 spectrometer (Horiba Ltd., Kyoto, Japan) was used to record the Fourier transformed infrared spectroscopy (FTIR) spectrum of nHAP at a wavenumber range of 400–4000 cm^−1^ with a resolution of 2 cm^−1^. The shape, size, and morphology of the microspheres and sintered scaffolds were observed through an inverted microscope (IX-71, Olympus Co., Tokyo, Japan) and a scanning electron microscope (SEM) (S-3000N, Hitachi Ltd., Tokyo, Japan) at 15 kV. The FTIR spectra of the V scaffold was compared with nHAP and the OC scaffold using a Horiba FT-730 spectrometer. Further confirmation on the incorporation of nHAP in OC scaffolds was determined through XRD analysis in comparison with V scaffolds and nHAP. In addition, presence of nHAP in C and OC scaffolds was tested through elemental analysis by an energy dispersive X-ray (EDX) analyzer (EX-250, Horiba Ltd., Kyoto, Japan).

Thermal characteristics of V and C scaffolds as well as the quantitative estimation of nHAP in C scaffolds were measured through thermogravimetric analysis (TGA) using TGA 2050 from TA instruments (New Castle, DE, USA). The samples were heated at a controlled rate of 10 °C/min from 25 to 700 °C and the resultant decomposition curve was plotted as residual weight (%) vs. temperature (°C).

Mechanical stability of the prepared V, C, and OC scaffolds was tested using an ElectroForce^®^ 5200 BioDynamic^®^ Test Instrument from Bose (Eden Prairie, MN, USA) equipped with a 250 N load cell. All samples were prepared in cylindrical shapes with diameter and height 5 mm each. A cross head speed of 5 mm/min was applied and the result was plotted as stress (MPa) vs. strain (%).

Surface characteristics of the V, C, and OC scaffolds were analyzed using a sessile drop contact angle goniometer (FTA125, First Ten Ångstroms, Portsmouth, VA, USA). Disc-shaped scaffolds with 1 cm diameter and 2 mm thickness were fixed on the sample holder. The wettability of the scaffold was evaluated by gently dripping a double-distilled water droplet on the surface. Respective images were recorded 2 s and 30 s and the contact angle was calculated by fitting a mathematical expression to the shape of the liquid drop and then calculating the slope of the tangent to the liquid drop at the liquid–solid–vapor interface line.

### 2.6. In Vitro Cell Culture Studies

#### 2.6.1. Cell Isolation and Expansion

Bone marrow stem cells (BMSCs) were isolated from New Zealand white rabbits following the procedures described previously [36]. 5 × 10^5^ BMSCs were re-suspended in 10 mL cell culture medium (DMEM medium containing 10% FBS and 1% penicillin-streptomycin) in a T75 culture flask and maintained at 37 °C in 5% CO_2_ atmosphere. The medium was changed every 2 days until reaching 80%–90% confluence. The supernatant medium was removed along with residual impurities followed by triplicate washing with phosphate-buffered saline (PBS). The adhered cells were detached using 0.05% trypsin–EDTA solution and transferred to a centrifuge tube. The tube was centrifuged for 5 min at 1000× *g* and 4 °C and the cell pellet was collected by removing the supernatant. The collected cell pellet was re-suspended in 10 mL cell culture medium and split into four T75 flasks. Cells at passage 3–4 were used for further studies. Chondrocytes were harvested from the knee articular cartilage of New Zealand white rabbits, as reported earlier [37]. The cells were sub-cultured in chondrocyte medium (DMEM/F12 supplemented with 10% FBS and 1% antibiotic/antimycotic) and passage two was selected for cell seeding.

#### 2.6.2. Cell Attachment and Morphology

Biocompatibility of the scaffolds was first tested through cell attachment using BMSCs. Cells were cultured on cylindrical shaped V, C, and OC scaffolds up to 28 days. Scaffolds sterilized by UV light were taken in a 24-well culture plate and pre-wet with DMEM followed by culturing with BMSCs in cell culture medium at a seeding density of 3 × 10^4^ cells/scaffold. Cells were seeded to scaffolds in both vertical and horizontal positions and cultured in 5% CO_2_ environment at 37 °C with regular replacement of fresh medium every two days. Morphology of the cells on the scaffold surface and interior was analyzed using a SEM (S-3000N, Hitachi Ltd., Tokyo, Japan).

#### 2.6.3. Mono-Culture with BMSCs and Chondrocytes

Qualitative assessment on the viability of adhered BMSCs and chondrocytes in C and V scaffolds were evaluated through a Live/Dead viability/cytotoxicity assay kit (Molecular Probes, Eugene, OR, USA). BMSCs were seeded in pre-wet cylindrical C scaffolds at a seeding density of 3 × 10^4^ cells/scaffold, whereas chondrocytes were seeded in V scaffolds at the same seeding density and cultured up to 28 days. C scaffolds were cultured in osteogenic medium (OM) (DMEM with 50 μΜ l-ascorbic acid phosphate, 0.1 μΜ dexamethasone, 10 mM glycerol 2-phosphate, 1% antibiotic-antimycotic, and 10% FBS), whereas V scaffolds were cultured in chondrocyte medium (DMEM/F12 supplemented with 10% FBS and 1% antibiotic-antimycotic). The culture medium was replaced with fresh medium every 2 days and washed with PBS prior to staining. The Live/Dead staining solution was prepared by mixing 2 µM calcein AM (excitation 494 nm and emission 517 nm for live cells) with 5 µM of ethidium homodimer-1 (EthD-1) (excitation 528 nm and emission 617 nm for dead cells) in culture medium. Samples were incubated with the staining solution at 37 °C for 30 min and imaged under a Zeiss LSM 510 Meta confocal laser scanning microscope (Carl Zeiss AG, Jena, Germany).

#### 2.6.4. Cell Proliferation

Cylindrical-shaped V, C, and OC scaffolds were sterilized by UV light for 4 h and placed in a 24-well culture plate. All scaffolds were pre-wet with DMEM followed by seeding with BMSCs at a density of 1 × 10^4^ cells/scaffold and the cells were allowed to adhere at 37 °C for 4 h. After the incubation period, the scaffolds were transferred to a new culture plate containing 1 mL OM and placed in a 37 °C humidified 5% CO_2_ incubator. The cell number was determined by DNA assay using Hoechst 33258 [38].

#### 2.6.5. Co-Culture with BMSCs and Chondrocytes

Cylindrical-shaped OC scaffolds with nHAP-containing bone part were designed to be cultured with BMSCs followed by culturing chondrocytes in the cartilage part. Briefly, the bone part of OC scaffolds was rinsed in OM followed by seeding with BMSCs (2 × 10^4^ cells/scaffold), and maintained in OM for 7, 14, and 21 days. At each time period, the OM was removed and the cartilage part of the OC scaffold was seeded with chondrocytes (1 × 10^4^ cells/scaffold) and maintained for another 7, 14, and 21 days in chondrocyte medium. Thus, the OC scaffold was immersed in OM and chondrocyte medium respectively before and after chondrocyte seeding in the cartilage part. The respective morphology of BMSCs and chondrocytes in the bone and cartilage parts of OC scaffolds were monitored through SEM. Morphology of BMSCs in the bone part of OC scaffolds after each co-culture time point was further evaluated through SEM observation to verify the cell behavior in bone part with additional culture in chondrocyte medium.

#### 2.6.6. Alizarin Red and Alcian Blue Staining

The effect of various stimulating factors in tissue-specific cell differentiation was analyzed through Alizarin red (AR) staining of the bone part and Alcian blue (AB) staining of the cartilage part. AR staining was done for both mono- and co-cultured samples, while AB staining was performed only for co-culture. In mono-culture, disc-shaped scaffolds (V, C, and OC) were seeded with BMSCs (1 × 10^4^ cells/scaffold) and cultured in normal medium (NM, DMEM with 10% FBS and 1% antibiotic-antimycotic) and OM. Acellular scaffolds were considered as controls for comparative evaluation towards bone formation. After 14 days culture in both NM and OM, samples were rinsed thrice with PBS and fixed with glutaraldehyde solution (2.5%) for 2 h. 0.5 g Alizarin red S (ARS) was dissolved in 25 mL deionized water adjusted to pH 4.1~4.3 with ammonium hydroxide to get the AR staining solution and each scaffold was immersed in 1 mL of the same, followed by incubation for 1 h at room temperature. Excess dye was washed off after incubation using deionized water, and the presence of mineral deposition was qualitatively evaluated by observing intensity of the red color on the scaffold surface. In co-culture, OC scaffolds were seeded with 1 × 10^4^ BMSCs in the bone part and cultured for 14 days (OM), followed by co-culturing with chondrocytes (1 × 10^4^ cells) in the cartilage part (chondrocyte medium) for 7 and 14 days. Scaffolds were separately analyzed through AR and AB staining by immersing the whole scaffold in 1 mL staining solution, as mentioned before. After incubation and washing, the intensity of the red and blue color on the bone and cartilage part was analyzed from photographs.

#### 2.6.7. Calcium Quantification

Calcium quantification from mineralization was measured through cetylpyridinium chloride (CPC) treatment. Scaffolds utilized for qualitative measurement of AR staining were washed with distilled deionized water and treated with 1 mL of 10% CPC solution for 1 h to chelate calcium ions. Both mono- and co-cultured samples were analyzed and the absorbance of the solution was read at 540 nm in an ELISA reader (OD_540_) (Synergy HTX, BioTek, Winooski, VT, USA) and normalized with cell number.

#### 2.6.8. Viability and Cytoskeletal Expression of Cells in Co-Culture

Viability of co-cultured BMSCs and chondrocytes in the bone and cartilage part of OC scaffolds were observed through confocal laser scanning microscope. Cell viability was tested through a Live/Dead viability/cytotoxicity assay kit. OC scaffolds pre-wet with DMEM was seeded with BMSCs at a seeding density of 2 × 10^4^ cells in the bone part and maintained in OM in a 24-well plate for two different time durations, viz: 14 and 21. Further, chondrocytes were seeded in the cartilage part (1 × 10^4^ cells) and maintained in chondrocyte medium for 7, 14, and 21 days. The scaffolds were washed with PBS, stained with the Live/Dead staining solution, and imaged for both bone and cartilage parts (excitation/emission 494/517 nm for live cells and 528/617 nm for dead cells) under a Zeiss LSM 510 Meta confocal laser scanning microscope. Cytoskeletal expression of BMSCs and chondrocytes in OC scaffolds were evaluated using phalloidin–tetramethylrhodamine B isothiocyanate (phalloidin–TRITC) (Sigma-Aldrich, St. Louis, MO, USA). The experimental design and time points for analysis were similar to Live/Dead staining. For cytoskeleton staining, cell-seeded OC scaffolds were treated with 4% formaldehyde in PBS for 20 min, followed by dehydration with ethanol and permeabilization with 0.1% Triton X-100 for 5 min. Cytoskeleton was stained with 50 µg/mL phalloidin–TRITC solution for 30 min at room temperature and then washed with PBS to remove unreacted phalloidin conjugates. Nuclear staining was done with (4′,6-diamidino-2-phenylindole) (DAPI) (KPL, Gaithersburg, MD, USA). Fluorescent images were recorded under a LSM 510 Meta confocal laser scanning microscope at an excitation/emission wavelength of 540/570 nm for phalloidin and 360/460 nm for DAPI.

#### 2.6.9. Immunofluorescent Staining of COL I, OCN, and COL II

Immunofluorescent staining was done for co-cultured OC scaffolds to observe and compare the bone and cartilage-specific protein expression. Cylindrical OC scaffolds pre-wet with DMEM were seeded with BMSCs at a seeding density of 2 × 10^4^ cells in the bone part and maintained in OM for different culture durations from 7 to 28 days. Furthermore, OC scaffolds with 14-day-cultured BMSCs (in bone part) were co-cultured with chondrocytes in cartilage part at a seeding density of 1 × 10^4^ cells and maintained the whole scaffold in chondrocyte medium for another 21 days. Cells in the scaffold were fixed by immersing in 4% formaldehyde in PBS for 30 min followed by washing with PBST (PBS with 0.1% Tween 20) for 3 times, 15 min each. Nonspecific labeling was blocked by incubating with 1 mL of HyBlock 1-min Blocking Buffer^®^ (Goal Bio, Taipei, Taiwan) followed by washing with PBST. OC scaffolds were incubated in either type I collagen (COL I) primary antibody (1:200 in PBS, mouse monoclonal anti-collagen I, Abcam, Cambridge, MA, USA) or osteocalcin (OCN) primary antibody (1:200 in PBS, guinea pig polyclonal anti-osteocalcin, Cloud-Clone Co., Houston, TX, USA) for 1 h to analyze the bone formation in the BMSCs-seeded part. Type II collagen (COL II) primary antibody (1:200 in PBS, guinea pig polyclonal anti-collagen II, Cloud-Clone Co., USA) was used to identify the cartilage-specific protein generation. Each sample was rinsed in PBS for 20 min followed by incubating COL I-treated scaffolds in Cy3-conjugated goat anti-mouse IgG secondary antibody (Jacksons Laboratories, Bar Harbor, ME, USA), OCN-treated scaffolds in FITC-conjugated goat anti-guinea pig IgG secondary antibody (Jacksons Laboratories, USA), or COL II-treated scaffolds in FITC-conjugated goat anti-guinea pig IgG secondary antibody (Jacksons Laboratories, USA) for 1 h. Another PBST washing was given prior to the addition of DAPI for nuclear staining. The immunofluorescent-stained OC scaffolds were observed for COL I at day 7, 14, and 28, while OCN formation was evaluated at day 14, 21, and 28. COL II formation was tested at day 14 and 21. Samples were rinsed once more before imaging under a laser scanning confocal microscope.

### 2.7. In Vivo Animal Study

#### 2.7.1. Animal Implantation

All animal procedures were approved by the Institutional Animal Care and Use Committee of Chang Gung University (IACUC Approval No. CGU16-043). 6–8 weeks old female nude mice weighing 20–30 g purchased from the National Laboratory Animal Center (Taipei, Taiwan) were used for the in vivo implantation. Pre-sterilized disc-shaped OC scaffolds were rinsed with DMEM followed by seeding with BMSCs at a seeding density of 2 × 10^4^ cells in the bone part and maintained in OM for 14 days in a 24-well plate with replacement of fresh medium every two days. OC scaffolds were further seeded with chondrocytes in the cartilage part with a cell seeding density of 1 × 10^4^ cells and maintained in chondrocyte medium for another 4 h for cell attachment. Acellular cylindrical OC scaffolds were used as control. Prior to surgery, all the animals were anesthetized using a mixture of 7 mg/kg xylazine (Rompun^®^, Bayer, Leverkusen, Germany) and 140 mg/kg ketamine (Ketalar^®^, Hoffman-La Roche Ltd., Basel, Switzerland). The back sides of the animals were sterilized with 75% alcohol solution, two subcutaneous pockets on each side of the back of the animal were made and one acellular and three co-cultured OC scaffolds were implanted intra-muscularly. The animals were sacrificed 4, 8, and 12 weeks post-implantation using overdosed pentobarbital and implants were harvested for further analysis.

#### 2.7.2. Gross and Microscopic Observation

Samples harvested at week 4, 8, and 12 were gross evaluated in comparison with controls. Effects of implantation on scaffold morphology, cell adhesion, and cell spreading were further verified through SEM analysis. Harvested samples were cut through the center of bone–cartilage intersections, washed with PBS, and fixed in 10% formaldehyde for 4 h. After PBS washing, alcohol gradient was used to remove the water content from the sample and vacuum dried overnight. Scaffolds were fixed on the carbon tape-pasted aluminum stub with the cross-sectional area facing outwards. Morphology of the cell-seeded scaffolds was recorded in comparison with acellular ones using SEM.

#### 2.7.3. Immunofluorescent Staining

Samples harvested at 4, 8, and 12 weeks were immersed in 4% formaldehyde for 4 h followed by washing thrice with PBST. Surface adhered fibrous tissue layer was removed and scaffolds were cut through the bone–cartilage interface for separate immunofluorescent (IF) staining. Nonspecific labeling was blocked followed by washing with PBST. The bone part of the scaffold was incubated in OCN primary antibody while the cartilage part was treated with COL II primary antibody for 1 h. Both samples were rinsed in PBS for 20 min followed by incubation in FITC-conjugated goat anti-guinea pig IgG secondary antibody for 1 h. DAPI was used for nuclear staining. Both IF-stained scaffold parts were observed under a laser scanning confocal microscope for OCN and COL II formation in vivo.

#### 2.7.4. Histological Analysis

The harvested OC scaffolds were immersed in 10% formaldehyde at room temperature and dehydrated using alcohol gradient, followed by embedding in paraffin wax and sectioned into 10 μm thick tissue sections in glass slides. The slides were further heated in a 70 °C oven followed by immersing in xylene for 5 min. The process was repeated thrice. Samples were re-hydrated using alcohol gradient and finally with water. After 5 min treatment with Tris-buffered saline containing 0.1% Tween 20, the bone part of the tissue sections was subjected to hematoxylin and eosin (H&E), AR, Masson’s trichrome stains, while the cartilage part was stained with H&E and AB. The images were recorded under an inverted optical microscope (IX-71, Olympus Co., Tokyo, Japan).

### 2.8. Statistical Analysis

All data were reported as mean ± standard deviation (SD) and the one-way ANOVA LSD test was used to determine the significant differences. A value of *p* < 0.05 was considered statistically significant.

## 3. Results and Discussion

### 3.1. Microsphere Preparation and Scaffold Fabrication

PLGA microspheres with or without nHAP were prepared through the standard oil-in-water emulsion/solvent evaporation method, as reported earlier [35]. The experimental procedures are shown in Step 1 of Figure 1. The size of spheres was distributed in the range of 75–800 μm, among which 250–500 µm was selected as the ideal size range. Specifically, microspheres in this size range were chosen, as this range may provide optimal pore sizes to allow cellular infiltration and cell-to-cell interaction [39]. The maximum yield within this size range was obtained by parameter optimization during the preparation step. Figure 1A,B is the respective light microscopic and SEM images of the prepared PLGA microspheres with size range 250–500 µm without (left) and with (right) nHAP. The effective incorporation of nHAP can be verified through the radio-opaque nature of PLGA/nHAP microspheres (right) compared to the transparent nature of virgin PLGA microspheres (left) in Figure 1A. The surface morphology of the same was further characterized through SEM and shown in Figure 1B. PLGA/nHAP microspheres had relatively rough morphology (right) compared to PLGA microspheres (left), due to the presence of nHAP particles. The scaffold was fabricated from the microspheres, as depicted in Step 2 of Figure 1. Figure 1C indicates the gross views of scaffolds prepared in various shapes with respect to different required experimental procedures in vitro and in vivo. Due to the osteo-conductive nature, the incorporation on nHAP in the scaffold will enhance its osteogenic differentiation effect toward BMSCs, and thereby is expected to be a key scaffold material for bone formation. V and C denote the scaffolds made from PLGA microspheres and PLGA/nHAP microspheres, respectively. OC represents a biphasic integrated structure of cartilage and bone parts sintered together in a single scaffold. Red arrows in Figure 1C point out the interface region in the curve-edged rectangular, disc, and cylindrical-shaped OC scaffolds, with distinguishable separating zones for cartilage and bone parts, respectively. The bright white appearance of the bone part is due to the presence of nHAP in PLGA/nHAP microspheres while the transparent nature of the cartilage part is attributed to the absence of the same in PLGA microspheres. The capability to generate OC scaffolds in various shapes also demonstrates the flexibility of the microsphere-based biphasic scaffold fabrication method to meet the need in repairing complex osteochondral defects.

### 3.2. Scaffold Characterization

#### 3.2.1. Morphology of Spheres and Scaffolds

**Figure 2:**
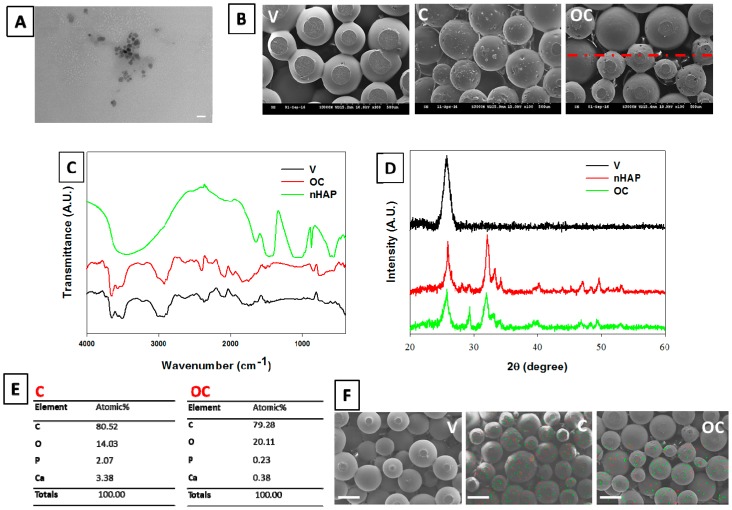
Physico-chemical properties of scaffolds. (**A**) TEM image of nHAP (bar = 20 nm); (**B**) SEM images of V, C, and OC scaffolds; (**C**,**D**) FTIR and XRD of nHAP and V and OC scaffolds; (**E**) EDX elemental atomic compositions of C and OC scaffolds; (**F**) elemental mapping of V, C, and OC scaffolds with Ca and P as green and red dots, respectively (bar = 300 μm). The red line in (**B**) indicates the interface region in the OC scaffold.

The prepared nHAP was characterized by TEM. The particles had a smooth round morphology with an average diameter of 15 ± 4 nm (Figure 2A). The morphology of sintered microspheres in V, C, and OC scaffolds is shown through cross-sectional SEM observation (Figure 2B). It is evident from the images that all microspheres showed uniform surface sintered to each other, making the scaffolds mechanically stable and macro-porous. The white dots in the C scaffolds further confirm the existence of nHAP in PLGA/nHAP microspheres after sintering [40]. The interface of OC scaffolds exhibits a rough surface textured with white-dotted PLGA/nHAP microspheres on one side of the image and positioned next to the relatively smoother PLGA microspheres on the other side. All scaffolds had a pore size ranging from 50 to 250 µm, making them ideal for cell penetration throughout the scaffolds [41].

#### 3.2.2. FTIR Analysis

nHAP and V and OC scaffolds were further characterized through FTIR (Figure 2C). The characteristic phosphate stretching vibrations were observed at 576, 612, and 930–1040 cm^−1^, whereas the stretching bands at 1510–1541 cm^−1^ confirm the presence of the hydroxyl group. The broad band observed at 3460–3560 cm^−1^ corresponds to the stretching mode of the hydroxyl group, which is characteristic of calcium phosphates such as nHAP [17]. PLGA exhibited characteristic alkyl group frequencies in the broad range 2800–2950 cm^−1^, hydroxyl vibrations at 3690, and 633 cm^−1^, C=O stretching at 1710 cm^−1^. There are also stretching bands due to asymmetric and symmetric C–C(=O)–O vibrations between 1105 and 1300 cm^−1^ [42]. The bands in these regions are useful in the characterization of esters. The phosphate bands at 578, 610, and 928 cm^−1^ in the OC scaffolds further confirm the presence of nHAP in the scaffold formed by sintered PLGA/nHAP.

#### 3.2.3. XRD Measurements

Crystallinity of nHAP was confirmed by XRD and compared with JCPDS-090432 (Figure 2D). Characteristic 2θ values of nHAP, V, and OC scaffolds were recorded and compared. nHAP showed prominent 2θ peaks at 32.2° (211 plane) and 25.9° (002 plane) with minor 2θ values at 29.3°, 33.2°, 34.3, 40.2°, 46.9°, 49.7°, and 53.3° [43]. V scaffolds had a single peak of PLGA at 25.7°, which is overlapping with the prominent nHAP peak. OC scaffolds exhibited the presence of the respective typical nHAP crystalline peaks with lowered peak intensities.

#### 3.2.4. Stoichiometric Analysis of nHAP by EDX

Apart from FTIR and XRD, nHAP content in C and OC scaffolds were cross-confirmed with EDX analysis. Figure 2E represents the EDX elemental compositions of the C and OC scaffolds. The presence of Ca and P peaks in both the C and OC scaffolds is evident, with the peak intensity of the OC being lower than that of the C, attributing to the lower overall nHAP content in the former. The scaffolds show a Ca/P stoichiometric ratio of 1.63 and 1.65 for the C and OC, respectively, in close relation with the theoretical value 1.67 for HAP [44]. Presence of nHAP in the C and OC scaffolds was further represented through elemental mapping, in which calcium was denoted as green dots and phosphate as red dots (Figure 2F). The V scaffolds did not show mapping colors contrast due to the absence of nHAP, while the C scaffolds showed color dots representing Ca and P throughout the scaffold. An interface mapping of the OC scaffolds revealed one part (lower half) of the scaffold is with Ca and P dots while the other without, confirming the distinguishable borderline between the bone and cartilage part of the OC scaffold.

#### 3.2.5. Thermal and Mechanical Analysis

**Figure 3:**
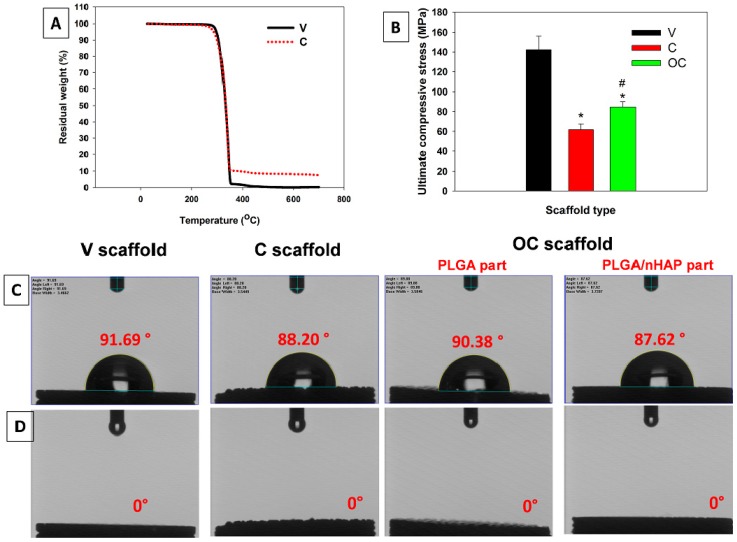
Thermal decomposition behavior (**A**) and mechanical testing (**B**) of V and C, and OC scaffolds. Surface hydrophilicity of V, C, and OC scaffolds by measuring water contact angles after 2 s (**C**) and 30 s (**D**); * *p*< 0.05 compared with V; # *p* < 0.05 compared with C.

The thermal properties of the V and C scaffolds were analyzed with TGA. As can be seen from Figure 3A, both scaffolds were thermally stable until ~274 °C from room temperature, without having any significant loss on the scaffold weight. The onset of weight loss was found at ~286 °C for both the V and C scaffolds. Further heating resulted in 50% weight loss at ~359 °C. The decomposition curve for both scaffolds was similar until reaching 376 °C, with a remaining weight of 12.3%. Further heating resulted in complete decomposition of the V scaffold at ~449 °C while the C scaffold had ~10% weight remaining. The residual weight percentage of nHAP-containing C scaffolds refers to the amount of nHAP present in the scaffolds due to the stability of ceramic particles, even at elevated temperatures. Most of the biodegradable polymeric materials are more prone to decomposition at higher temperatures, leading to complete charring prior to reaching 500 °C [34]. However, the ceramic counterparts of the same in the scaffold are much more stable, allowing it to retain residual weight during the thermal decomposition cycle at higher temperatures, thus accounting for the quantitative evaluation of nHAP in the C scaffolds.

Vigilant selection of materials in osteochondral tissue engineering is crucial, since supporting the substrate’s mechanical strength, structural support, and favorable culturing environment is critical during the early stages of the regenerative process. Optimal mechanical properties and suitable structures will ultimately promote bone–cartilage formation and implant healing. The V scaffolds had the maximum ultimate compressive strength of 142 ± 14 MPa, which is significantly higher than that of the C (62 ± 6 MPa) and OC (85 ± 5 MPa) scaffolds (Figure 3B). The median compressive strength value for the OC scaffolds should be due to the combination of PLGA and PLGA/nHAP microspheres in a single construct, accounting for the combinatory effect of both V and C scaffolds, which are made from PLGA and PLGA/nHAP microspheres separately. Generally, incorporation of an optimum percentage nHAP should increase the mechanical strength of the C scaffolds [45]. The addition of nHAP particles to PLGA microspheres may result in a reduction in mechanical strength of C compared to V scaffolds. Though the percentage of nHAP in PLGA/nHAP microspheres is ~10%, the irony of reduction in compressive strength refers to the sintering aspects of scaffolds. The sintering mechanism of scaffolds is through the surface melting of compressed microspheres in the heating cycle and permanent adhering of the same during the cooling cycle. Since PLGA/nHAP microspheres had uniformly distributed nHAP on the surface, the thermal resistibility of the same would have interfered with the surface melting–adhering mechanism during the heat sintering process, thereby reducing the mechanical strength of the C scaffolds. More studies on the time–temperature dependence of the scaffold’s mechanical strength are necessary to elucidate this aspect.

#### 3.2.6. Contact Angle Measurements

Contact angle is a measure of hydrophobicity or surface wettability of a material. The higher the contact angle values, the higher the hydrophobicity of the material is. The goniometer was used here to measure the hydrophobicity of the scaffolds based on pictures of a water droplet on the scaffold surface. Here, the contact angle was measured at two time points, 2 s (Figure 3C) and 30 s (Figure 3D) after placing the drop on the scaffold surface. The V and C scaffolds displayed a contact angle of 91.69° and 88.20°, respectively, showing their less hydrophilic nature, similar to previous reports [46]. The OC parts fabricated from PLGA and PLGA/nHAP microspheres also showed similar values at 90.38° and 87.62°, respectively, revealing that different microspheres in different parts of the OC scaffold maintain its hydrophilicity after the physical sintering process. While considering the shape of the water droplet after 30 s, the contact angle was zero, or none of the scaffolds showed the existence of a water droplet on top of the scaffold surface. This could be attributed to the porosity of microspheres rather than wettability. Since all scaffolds have large pore sizes between microspheres (Figure 2B), drops could easily be absorbed into the scaffold interior through gravitational force and thereby rapidly disappear from the scaffold surface. Contact angle measurements confirm that though the surface characteristic is less hydrophilic in nature, faster absorption of water drops after 30 s indicates the high porosity of scaffolds, which is one of the essential requirements for an ideal scaffold for tissue regeneration.

### 3.3. In Vitro Cell Culture Studies

#### 3.3.1. Cell Morphology

**Figure 4:**
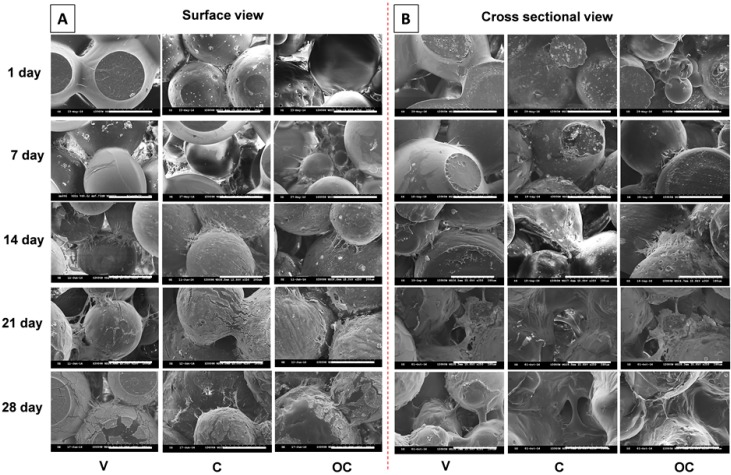
Cell infiltration and cell morphology of BMSCs (bone marrow stem cells) in V, C, and OC scaffolds. The increased cell density and spreading from day 1 to day 28 from SEM images of scaffold surface (**A**) and cross section (**B**) validates the biocompatibility and pore interconnectivity in all scaffolds. Bar = 200 μm

The use of mesenchymal stem cells on biodegradable implants has been shown to be effective for repairing osteochondral defects in vivo [47]. Also, many results report the added advantages of using two different cell types to regenerate osteochondral defects [40]. In this work, we first analyzed the ability of the OC scaffolds to successfully support BMSCs attachment and proliferation in vitro, in comparison with V and C scaffolds. When treating osteochondral defects, the “bone-like” layer should aim at the repair of the cortical-subchondral bone, while the “cartilage-like layer” must provide a good environment for cartilage regeneration. The effect of microsphere surface characteristics and interior pore gap on cell adhesion, spreading, and cell–cell interconnectivity was analyzed through surface (Figure 4A) and cross-sectional (Figure 4B) SEM observations. Though the intended use was for osteochondral repair, the cell behavior was studied using only BMSCs to verify the general cell characteristics such as adhesion and spreading and also to evaluate the ability of the sintered microsphere structure to support cell growth and spreading without obstructing the scaffold pores, which are the key factors when considering waste and nutrient transport in the scaffold. At day 1, cells attached well to both surface and interior sintered microspheres of all scaffolds, albeit with low cell density and spreading. Cell morphology at day 7 was much altered, where cells started spreading more on the sphere-to-sphere intersections by outspreading filopodial extensions regardless of scaffold type. Much robust cell behavior was observed at day 14, where cells extended their growth on the wall of the microsphere, giving a thin cellular coating on the sphere surface. The cell density in the interstitial space was much higher than at previous time points, as observed from both surface and cross-sectional images. The cell density was significantly higher than earlier culture duration at day 21 and 28. At these points, cells became more flattened, cellular wall density on microsphere surfaces became much denser, and cells connected adjacent spheres through filopodial extension, as evidenced from the images. The cross-sectional image indicates that cells were able to penetrate deeper into the scaffold pores, which is an important observation, since a good cell distribution within the whole scaffold prominently affects the overall performance of the construct, without limiting the diffusion of nutrients and oxygen. Though all scaffolds exhibit similar characteristics in terms of cell interactions, we speculate that this result is quite promising since it validates that the microsphere-based scaffolds possess an adequate pore size and pore distribution to effectively allow cells to adhere and maintain their functions.

#### 3.3.2. Viability of BMSCs and Chondrocytes in Mono-Culture

**Figure 5:**
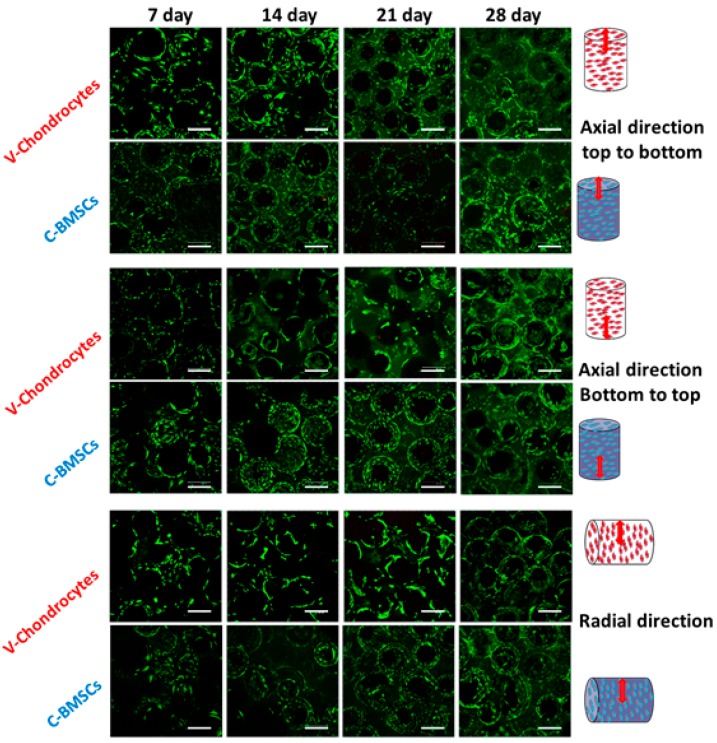
Live/Dead assays of mono-cultured chondrocytes in V scaffolds and BMSCs in C scaffolds. Confocal laser scanning microscope images from axial and radial directions at day 7, 14, 21, and 28 indicate the spread nature and uniform distribution of both cells. Bar = 300 μm.

The viability of cells in scaffolds was evaluated using a Live/Dead assay kit that shows cell morphology and distribution in the scaffold interior after imaged by confocal microscopy (Figure 5). As mentioned before, the behavior of cells in the OC scaffolds was evaluated using chondrocytes and BMSCs for the V and C scaffolds separately, using chondrocyte and osteogenic medium. Due to the inability of a laser scanning confocal microscope to have high depth scanning arrays for the scaffolds, scaffolds were scanned both axially (from the top or the bottom of scaffolds) and radially to determine cell distribution in 3D within the scaffold. Uniform cell distribution and high cell viability in both the V and C scaffolds could be confirmed from the green fluorescence signal of live cells and few red signals from dead cells. The cell density is lower at day 7 than day 14, irrespective of the scaffold type or the scan direction. Cell morphology at day 21 and 28 were almost similar to that at day 14, which can be correlated with the flattened cell morphology on the spherical surface of the microsphere, as shown from the SEM images in Figure 4. Unlike other types of scaffolds, cells in a microsphere-based scaffold adhere first, spread on the microsphere wall, and then deposit as a monolayer over the surface of the microsphere in the early stages, followed by thickening of the cellular layer as reported previously [48]. A few dark empty spaces inside cellular circles are due to the out-of-focus plane of different microspheres inside the scaffold. However, no significant difference in cell morphology was observed among chondrocytes-seeded V scaffolds or BMSCs-seeded C scaffolds at any point of the culture period. Nevertheless, cells grew well and proliferated around the microspheres and throughout the scaffold, cross-confirming the advantage of the macroporous structure of the scaffolds for tissue regeneration in vitro.

#### 3.3.3. Cell Proliferation

**Figure 6:**
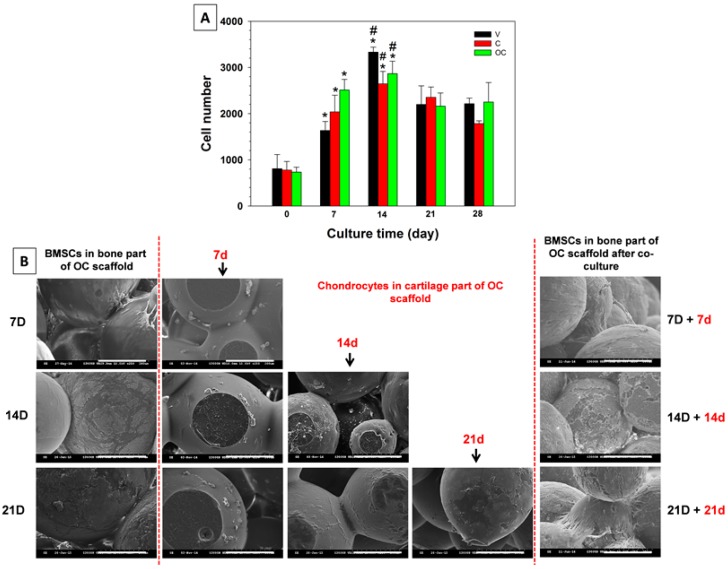
(**A**) Cell numbers of BMSCs from DNA assays when cultured in in V, C, and OC scaffolds up to 28 days. * *p* < 0.05 compared with day 0, ^#^ *p* < 0.05 compared with day 7 for each scaffold; (**B**) SEM images of the morphologies of BMSCs in the bone part of OC scaffolds before and after co-culture with chondrocytes. 7 D, 14 D, and 21 D represents the morphology of mono-cultured BMSCs in the bone part for 7, 14, and 21 days, while 7 d, 14 d, and 21 d is the morphology of co-cultured chondrocytes in cartilage part of OC scaffolds after 7, 14, and 21 days. 7 D + 7 d, 14 D + 14 d, and 21 D + 21 d denote the morphologies of BMSCs in the bone part of OC scaffolds after immersing in chondrocyte medium for another 7, 14, and 21 days during co-culture. Bar = 200 μm.

Cell proliferation in scaffolds was evaluated through DNA contents (Figure 6A). The cell number after 4 h of attachment (day 0) was taken as the reference point to be compared with later periods of cell proliferation of BMSCs in V, C, and OC scaffolds. All scaffolds exhibited an increase in cell number after day 7, compared to day 0 and then reached a maximum at day 14. There was no significant increase in cell number following day 14, ascribed to the differentiation of BMSCs in OM to osteoblasts with reduced cell proliferation rate. Our previous studies also confirmed the same trend of stem cells differentiation in osteoconductive-inductive environments [34]. The rationale behind the choice of BMSCs alone for cell proliferation rather than using both cell types is based on two factors: in vitro co-culture and in vivo aspects. In the former case, since V and C scaffolds are separately compared here with OC scaffolds, mono-culture strategy is practically more significant to evaluate the cell proliferation and differentiation potential. Though the V part is assigned for chondrocytes and the C part for BMSCs, culturing two different cell types in OC scaffolds at two different time points and further with cell number evaluation is practically irrelevant. Moreover, most of the in vitro co-culture experiments performed throughout this study was by seeding BMSCs in the bone part for pre-optimized times, followed by seeding chondrocytes in the cartilage part and maintaining the culture for another two or three weeks, which only aimed at examining the potential of both cell types to maintain their phenotype in co-culture conditions. Considering the in vivo aspect, OC scaffolds were designed to culture with BMSCs in the bone part for the first 14 days, followed by seeding with chondrocytes in the cartilage part for 4 h prior to implantation, denoting the non-requisite strategy of co-culture in cell proliferation studies. Further, the logic behind the culture of V scaffolds with BMSCs rather than chondrocytes is to validate the effect of osteo-conductive nHAP in C and OC scaffolds towards stem cell differentiation.

#### 3.3.4. Co-Culture with BMSCs and Chondrocytes

Bi-phasic tissue engineering always has the complexity of recognizing proper cues toward preferred cell differentiation and thereby develops separate tissues. Co-culture offers the benefits of distinguished regeneration and faster development to multiple tissue types compared with mono-culture. Figure 6B shows the SEM images of morphologies of the BMSCs-seeded bone part and chondrocytes-seeded cartilage part of the OC scaffolds at various co-culture durations. The left panel represents the BMSCs morphology in the bone part in OM at day 7, 14, and 21. Scaffolds seeded with BMSCs in the bone part for 7 d had similar cell adhesion patterns as observed before in Figure 4, while the morphology of the chondrocytes-grown cartilage part for another 7 d had a relatively more round morphology, as shown in the middle panel (red letters). The extent of cell spreading and flattening increased with time for both cell types. The morphology of chondrocytes at each co-culture time point was similar, irrespective of BMSCs culture durations in the bone part. Similar to BMSCs, chondrocytes spread well and flattened throughout the scaffolds with respect to co-culture durations, with the maximum cell density at day 21, which could be correlated with the fluorescently stained chondrocyte morphology shown in Figure 5. However, after co-culture, it is necessary to prove the existence of BMSCs with no alteration in cell morphology. The right panel is the SEM images of BMSCs in the bone part after co-culture with chondrocytes. The 7 D + 7 d represents the morphology of BMSCs cultured in the bone part in OM 7 days, followed by immersing in chondrocyte medium for another 7 days while co-culturing. In a similar aspect, 14 D + 14 d and 21 D + 21 d SEM images respectively represent the morphology of BMSCs after 28 days (i.e., 14 days +14 days) and 42 days (i.e., 21 days + 21 days) BMSCs/chondrocyte co-culture durations. Though BMSCs in the bone part have undergone more culture time in chondrocyte medium during co-culture, the cell morphology of BMSCs confirms cell survival/proliferation was not affected by co-culture. Only a few reports are available on the successful regeneration of biphasic tissues from mono-culture using a single cell source such as BMSCs, due to the difficulty of maintaining distinguished environments in a single scaffold. Studies are not satisfactory to establish an ideal scaffold design with separate zones for loading specific growth factors/proteins, which could lead to separate tissues such as bone and cartilage. The restriction of specific growth factors/proteins eluted from the scaffold to be within a specific region of the environment that BMSCs are exposed to during co-culture is considerably challenging [49]. Our scaffold design without eluting growth factors/proteins but with osteo-conductive nHAP in the bone part and co-cultured chondrocytes as the cartilage part is very promising.

#### 3.3.5. Qualitative Evaluation on Bone–Cartilage Formation in Vitro

**Figure 7:**
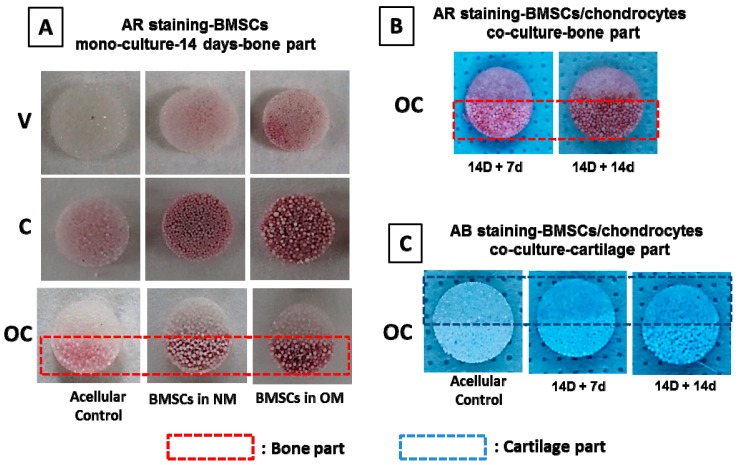
(**A**) The Alizarin red (AR) staining images of acellular control and BMSCs-seeded V, C, and OC scaffolds after mono-culture in normal medium (NM) and osteogenic medium (OM) for 14 days; (**B**,**C**) are the Alizarin red (AR) and Alcian blue (AB) staining images of BMSCs in the bone part and chondrocytes in the cartilage part of OC scaffolds. 14 D + 7 d and 14 D + 14 d denote the culture of BMSCs in the bone part of OC scaffolds in OM for 14 days followed by immersing in chondrocyte medium for another 7 and 14 days during co-culture.

Figure 7A,B demonstrate positive staining for Alizarin red (AR), while Figure 7C depicts the positive staining for Alcian blue (AB). The AR stain evaluates calcium deposits in the mineralized extracellular matrix (ECM) of differentiated BMSCs when calcium forms an Alizarin red S-calcium complex in a chelation process and the degree of mineralization will be directly proportional to the intensity of the stain. Considering the cell proliferation, the duration of BMSCs mono-culture in the bone part of OC scaffolds was selected at 14 days. The effect of nHAP alone in C scaffolds and the bone part of the OC scaffolds towards BMSCs differentiation was tested through mono-culture in normal medium (NM) and osteo-inductive medium (OM). Both the C scaffolds and bone part of the OC scaffolds displayed a significant increase in AR stain intensity due to the mineralization of BMSCs. These results are supportive of the fact that nHAP particles in the C and OC scaffolds, due to its osteo-conductive nature [50,51], which triggered BMSCs differentiation along an osteogenic pathway, even in the absence of soluble growth factors in NM. However, nHAP alone is not enough to guarantee osteoblast differentiation. Therefore, the combined effects of osteo-conductive nHAP and osteogenic inducing factors were evaluated through BMSCs cultured in OM. That the maximum stain intensity was observed in the C and OC scaffolds announces the synergistic effect of nHAP and inducing factors on commitment of BMSCs toward the osteogenic lineage in OM [52,53]. To eliminate the possibility that positive staining was due to the calcium ions in nHAP within the scaffolds, AR staining was also performed on acellular control scaffolds, which showed much less stain intensity compared to BMSCs-seeded scaffolds. The results demonstrate that BMSCs mono-culture in the presence of osteogenic factors could lead to a dramatic increase in mineral deposition, which further leads to bone formation. Based on mono-culture results, OC scaffolds were seeded with BMSCs in the bone part and cultured in OM for 14 days, followed by chondrocytes seeding in the cartilage part for 7 and 14 days (Figure 7B). As expected, the 14-day (14 D + 14 d) co-cultured scaffolds had maximum AR stain intensity in the bone part compared to the 7-day (14 D + 7 d) co-cultured samples, indicating continued mineral deposition of BMSCs even in chondrocyte medium, due to the presence of osteo-conductive nHAP.

Alcian blue is any member of a family of polyvalent basic dyes and is used to stain acidic polysaccharides such as glycosaminoglycans (GAGs) in cartilages. Therefore, AB stain could demonstrate that chondrocytes were able to deposit a GAGs-rich ECM in the cartilage part of OC scaffolds. Similar to AR staining, OC scaffold having pre-differentiated BMSCs in the bone part was co-cultured with chondrocytes in the cartilage part for 7 days (14 D + 7 d) and 14 days (14 D + 14 d), followed by AB staining of the whole scaffold. That positive AB staining was only observed in the cartilage part of OC scaffolds at both time points confirms the maintenance of chondrogenic phenotype of seeded chondrocytes during co-culture and cartilage formation in vitro (Figure 7C). False positive AB staining could also be discounted through AB staining on acellular control scaffolds. Thus, the qualitative measurements of bone–cartilage formation in OC scaffolds through AR–AB stains endorse the potential of the same in osteochondral regeneration.

#### 3.3.6. Calcium Quantification

**Figure 8:**
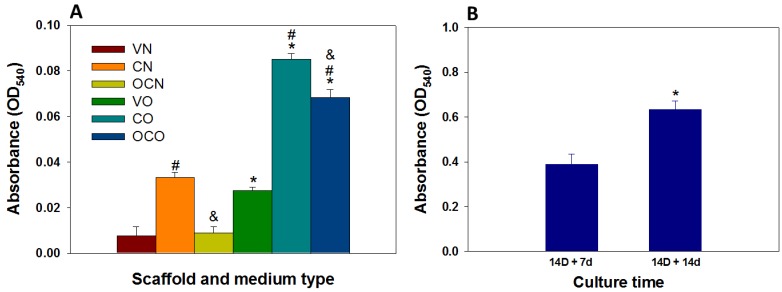
(**A**) Quantitative estimation of calcium deposition in V, C, and OC scaffolds after mono-culture of BMSCs in different scaffolds in normal medium (NM) and osteogenic medium (OM) for 14 days. VN, CN, and OCN denote V, C, and OC scaffolds in NM whereas VO, CO, and OCO denote V, C, and OC scaffolds in OM. * *p* < 0.05 compared with NM for each scaffold, ^#^ *p* < 0.05 compared with V for each medium, ^&^ *p* < 0.05 compared with C for each medium; (**B**) Total calcium deposition in the bone part of co-cultured BMSCs and chondrocytes in OC scaffolds. 14 D + 7 d denotes 14 days in osteogenic medium and 7 days in chondrocyte medium; 14 D + 14 d denotes 14 days in osteogenic medium and 14 days in chondrocyte medium. * *p* < 0.05.

Positive AR staining from gross view observation (Figure 7A,B) is the evidence for BMSCs mineralization. However, calcium quantification with cetylpyridinium chloride (CPC) could estimate the extent of calcium-based mineral deposits by BMSCs. When scaffolds react with CPC, it extracts the ARS bound to Ca^2+^ on the cell surface and the absorbance of the extracted solution at 540 nm will provide direct estimation of the extent of BMSCs mineralization. Figure 8A represents the calcium quantification of BMSCs mono-cultured in V, C, and OC scaffolds for 14 days whereas Figure 8B denotes those co-cultured in OC scaffolds. AR-stained V, C, and OC scaffolds mono-cultured with BMSCs in NM is denoted as VN, CN, and OCN while the same construct cultured in OM was termed VO, CO, and OCO. Considering the same scaffold, the absorbance values in NM show the least reading while in OM it was much higher. However, C scaffolds in NM had a relatively higher absorbance value than V in OM, probably due to the binding of ARS to nHAP in the scaffold. That OC scaffolds had a higher absorbance value than V scaffolds but lower than C scaffolds in OM indicates the synergistic effect of nHAP and osteogenic inductive factors in OM in enhancing calcium deposition, in accordance with the qualitative AR staining results of BMSCs mono-culture shown in Figure 7A.

Besides mono-culture, OC scaffolds co-cultured with BMSCs and chondrocytes were further investigated for calcium content. OC scaffolds with 14 days pre-differentiated BMSCs in the bone part, co-cultured with chondrocytes in the cartilage part for 7 and 14 days are denoted as 14 D + 7 d and 14 D + 14 d, respectively, in Figure 8B. The absorbance values observed for 14 D + 7 d was lower compared to 14 D + 14 d, indicating continued differentiation of BMSCs in chondrocyte medium after the commitment of BMSCs toward the osteogenic lineage during the initial culture in OM. Furthermore, the calcium content of the 14 D + 7 d co-culture group is much higher than that of any of the BMSCs mono-culture groups, due to the prolonged culture duration of 21 d. This culture duration is longer than the culture time (14 days) required for the maximum cell number (Figure 6A), which is critical in continued differentiation and mineralization of BMSCs. Since the calcium deposition of stem cells starts at later stages of cell proliferation, duration of culture period is very critical in the extent of calcium deposition [34,54]. Indeed, these results can be correlated with the cell proliferation data shown in Figure 6A, claiming the maximum cell number at day 14, followed by a steady value on the remaining days. This confirms the differentiation of BMSC to osteoblasts in the later stages of cell proliferation and thereby accounts for the higher calcium deposition for co-culture of BMSCs and chondrocytes in OC scaffolds. The results are also convincing for the fact that BMSCs are not influenced by the presence of the chondrocyte medium used for co-culture, but continue their mineralization and maintain their phenotype throughout the in vitro culture period.

#### 3.3.7. Viability and Morphology of BMSCs and Chondrocytes during Co-Culture

**Figure 9:**
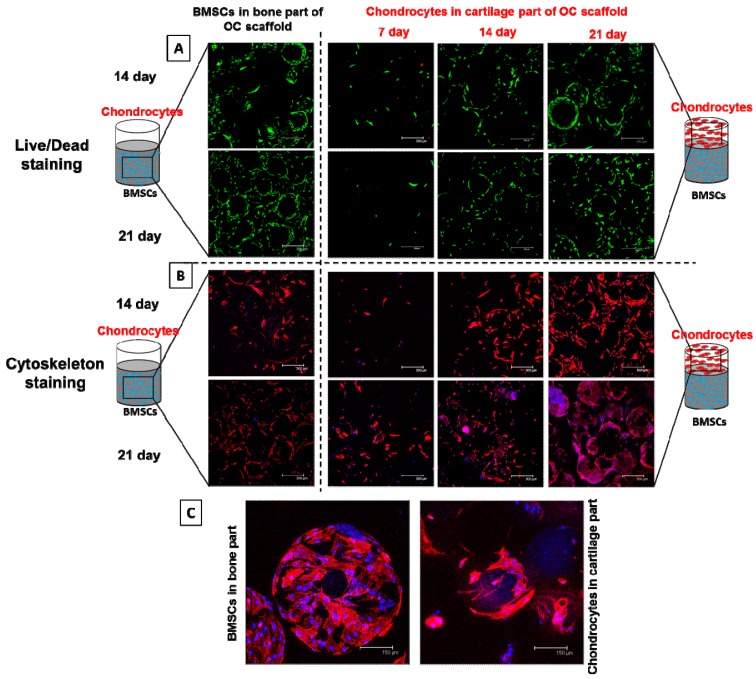
Live/Dead cell assays (**A**) and cytoskeletal arrangement determined by phalloidin/DAPI staining (**B**) of co-cultured BMSCs and chondrocytes in OC scaffolds. Bar = 300 μm; (**C**) The cytoskeletal arrangement of BMSCs and chondrocytes on a single microsphere surface in the bone and cartilage part at day 14. Bar = 150 μm.

Apart from mono-culture, viability of BMSCs and chondrocytes during co-culture was evaluated through Live/Dead assays (Figure 9A). As in mono-culture, the absence of red signals demonstrates the high viability of both BMSCs and chondrocytes during co-culture. BMSCs cultured for the initial 14 and 21 days had no distinguishable difference in cell morphology as observed for mono-culture in Figure 5. Chondrocytes exhibited comparable cell morphology in the cartilage part of OC scaffolds at each time point, irrespective of BMSCs culture duration. Similar to BMSCs, density of viable cells was the maximum for chondrocytes at day 21 of co-culture, indicating the high survival rate of the same. The densely populated live chondrocytes at day 21 indicates the cartilage part of OC scaffolds provides a favorable environment for cartilage development, while comparable cell densities at the same time point for the BMSCs-seeded bone part endorses the favorable cellular response of the OC scaffolds to maintain cell viability and support growth of BMSCs during co-culture for bone development. SEM images of the morphology of BMSCs in the bone part of OC scaffolds (Figure 6B) after various durations of chondrocyte co-culture cross-confirm the ability of BMSC to maintain the spread morphology.

The morphology of co-cultured cells on microspheres of OC scaffolds was validated through staining of the actin cytoskeleton of adhered cells. The cytoskeleton plays important roles in cell morphology, adhesion, growth, and signaling [55]. Changes in the cytoskeleton of the cell allow the cell to migrate, divide, and maintain its shape. The cytoskeleton consists of three components: actin filaments, intermediate filaments, and microtubules. The backbone of the cytoskeleton is F-actin, which clusters to form actin filaments. As explained before, unlike other scaffolds, cells on microspheres adhere first, spread on the microsphere wall, and make a monolayer over the microsphere in the early stages of cell adhesion. However, in the later stages, cells preferentially grow around the gaps among the microspheres, fill the gaps, and connect each adjacent sphere through a cellular bridging [56]. Being an abundant protein in cells, actin staining could help to visualize the migration and 3D proliferation of the cells within the scaffold structure. The versatility of the confocal microscope to scan various planes and their corresponding maximum projection would provide a better understanding of cellular morphology throughout the matrix. Figure 9B represents the confocal images of rhodamine-phalloidin stained cytoskeletal actin filaments of BMSCs and chondrocytes in bone and cartilage parts of OC scaffolds, respectively. Similar to Live/Dead staining, the intensity of actin staining of differentiated BMSCs in the bone part of OC scaffolds was almost similar at day 14 and 21, confirming the possible transformation of BMSCs to osteoblast differentiation. Considering the cartilage part at different co-culture times, chondrocytes had minimum stain intensity at day 7, enhanced at day 14, and reached the maximum at day 21, confirming the same trend of cell proliferation. Both cells almost covered the microsphere gaps and surface at later stages of culture. The cell spreading on a single microsphere surface is shown in the enlarged microscopic images in Figure 9C. Since both cells attained the shapes of the microspheres they adhered, actin staining was not a conclusive tool to declare the morphological difference between cells in bone and cartilage parts. More detailed biochemical assays are mandatory to reveal the efficiency of osteo-conductive/inductive factors and the BMSCs-chondrocytes approach towards osteochondral regeneration.

#### 3.3.8. Immunofluorescent Staining of Bone- and Cartilage-Specific Proteins

**Figure 10:**
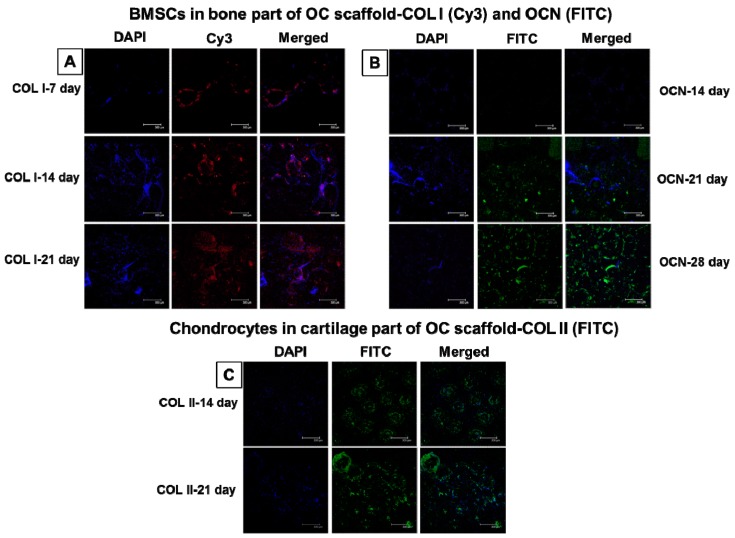
Immunofluorescent staining images using DAPI for cell nucleus and Cy3-conjugated antibody for type I collagen (COL I) (**A**); DAPI for cell nucleus and FITC-conjugated antibody for osteocalcin (OCN) (**B**); DAPI for cell nucleus and FITC-conjugated antibody for type II collagen (COL II) (**C**) at various durations of BMSCs-chondrocytes co-culture in OC scaffolds. Bar = 300 μm.

Figure 10A,B reveals time-lapsed IF staining of bone-specific proteins COL I and OCN in the bone part during co-culture. Figure 10C is the corresponding IF staining of cartilage-specific protein COL II in the cartilage part during co-culture. COL I production in the bone part of OC scaffolds was on day 7, 14, and 21 in contrast to day 14, 21, and 28 for OCN, as COL I and OCN are respectively the early and late markers of osteogenic differentiation. The blue fluorescent images represent DAPI-stained nucleus, red-colored Cy3 shows COL I in the ECM, and merged images are the combination of DAPI and Cy3 (Figure 10A). The production of COL I was minimum at day 7 compared to day 14, judging from the staining intensity, while day 21 had almost similar COL II protein expression to day 14. The relatively similar COL I protein production at day 14 and 21 for BMSCs cultured in the bone part of OC scaffolds is validated through the early stage COL I expression pattern of BMSCs differentiation [54,57]. The well spread appearance of Cy3-conjugated COL I at later stages of observation also confirms the uniform protein distribution within the ECM.

Bone formation in OC scaffolds was further confirmed through OCN protein expression, as shown in Figure 10B. Unlike COL I, OCN had almost no expression at day 14 (shown in green), though the DAPI-stained cell nucleus was present. However, the expression of FITC-conjugated OCN present in the ECM was higher at day 21, which further enhanced to a maximum at day 28. Being a late expression protein marker during bone formation, OCN displayed the same trend as reported in our previous studies [34,54]. Figure 10C demonstrates the protein expression of COL II in the cartilage part of OC scaffolds during co-culture. Similar to OCN, positive COL II staining using FITC-conjugated antibody confirmed cartilage formation in the cartilage part of OC scaffolds [58]. DAPI-stained cell nucleus in the well distributed COL II confirms the proliferation of chondrocytes during the regeneration of cartilage in the cartilage part of OC scaffolds, leading to the formation of osteochondral tissue. The green signal from FITC-conjugated secondary antibody indicates COL II distribution is more spherical in shape along the contour of a microsphere at day 14, whereas it is well distributed throughout the scaffold at day 21, confirming the expansion of chondrocyte ECM towards the pores within the scaffold. Actin-stained chondrocyte morphology in co-cultured OC scaffolds (Figure 9B) could be compared with the current observations in Figure 10C, confirming the attachment of chondrocytes over the microsphere surface at day 14 but extending cell deposition to the pores at day 21, similar to the COL II expression. The results are conclusive of the fact that the BMSCs-seeded bone part and chondrocyte-seeded cartilage part of OC scaffolds showed a great extent of bone and cartilage formation in vitro.

Other than difference in material composition in OC scaffolds employed here, it is also feasible to develop PLGA microsphere-based scaffolds with continuous gradients of bioactive cues [59]. The OC scaffold can be designed with opposing gradients of chondrogenic microspheres (encapsulating transforming growth factor-β1) and osteogenic microspheres (encapsulating bone morphogenetic protein-2) to direct the differentiation of BMSCs regionally toward osteogenesis and chondrogenesis [60].

### 3.4. In Vivo Animal Studies

#### 3.4.1. Gross and Microscopic Evaluation

**Figure 11:**
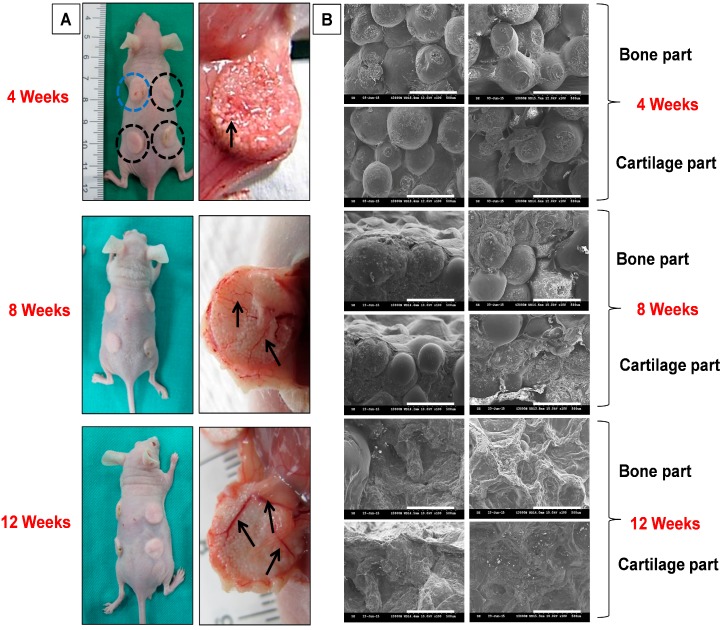
The gross views (**A**) and SEM images (**B**) of acellular OC scaffolds (control) and cell-seeded OC scaffolds (sample) harvested from nude mice 4, 8, and 12 weeks post-implantation. The blue and black circles indicate the control and the sample, respectively. Black arrows indicate blood vessel formation on scaffolds in (**A**); BMSCs and chondrocytes were co-cultured in OC scaffolds and bone and cartilage parts of scaffolds are observed by SEM in (**B**). Bar = 500 μm.

For in vivo studies, subcutaneous implantation in nude mice was used to evaluate the biocompatibility of acellular OC scaffolds (control) and cell-seeded OC scaffolds constructs (sample) and their potential in ectopic osteochondral tissue formation. Figure 11A is the gross view photographic observations of harvested controls and samples 4, 8, and 12 weeks post-implantation. Both bone and cartilage parts had blood vessel formation on the surface and interiors of the scaffold (black arrows) at all time points with more intense vascularization after week 12. The shape of the scaffolds was found to be slightly deforming with harvesting times, due to in vivo degradation. Moreover, mice fibrous tissue layer adhesions on scaffold peripherals were observed, which might be due to the high porosity of OC scaffolds. The cross-sectional SEM imaging was performed to observe the cells inside the scaffold at each time point (Figure 11B). A very dense population of sphere-bound cells was observed in both bone and cartilage parts of the OC scaffolds after 4 weeks. In both cases, most of the cells were bridging the gap between adjacent microspheres and thereby filling the porous intersections. Control samples had few cells adhered to microspheres due to fibroblasts infiltration from the host tissue. Both control and sample groups showed unchanged microsphere morphology, confirming their physical stability for short in vivo durations. A dramatic variation in scaffold morphology was observed at week 8, where bone and cartilage parts were surrounded with cellular layers of differentiated BMSCs and chondrocytes, respectively. A few spheres had completely disappeared or covered with newly-formed tissue. The sphere shape deformation was more at 8 weeks compared to week 4, suggesting initiation of bio-degradation. Control samples still had some host cell infiltration similar to 4 weeks, but negligible in comparison with bone and cartilage parts of cell-seeded scaffolds. A complete cellular regeneration was observed on both parts of the OC scaffold after 12 weeks of implantation. Microspheres were partially or completely degraded and cellular multilayers replaced the porous intersections of the scaffold. The narrow cellular bridging observed at 4 and 8 weeks re-formed to thick intense tissue channels at week 12, confirming the faster regeneration of osteochondral tissue. Unlike previous observations, control samples at 12 week displayed a complete collapse of spherical integrity within the scaffold, showing no signs of regeneration. Thus, the microscopic observations of implanted OC scaffolds assure the regeneration potential of co-cultured OC scaffolds in vivo.

#### 3.4.2. Immunofluorescent Staining for Bone and Cartilage Formation in vivo

**Figure 12:**
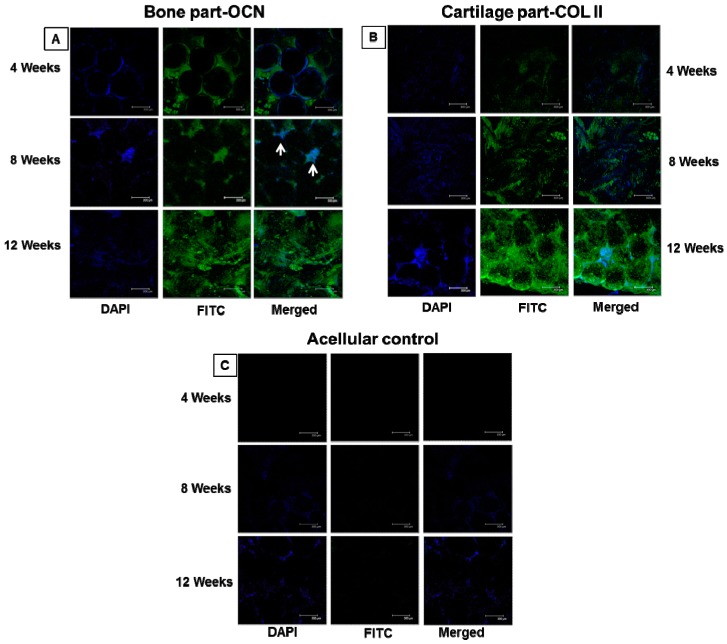
Immunofluorescent staining images using DAPI for cell nucleus and FITC-conjugated antibody for osteocalcin (OCN) in the bone part (**A**) and DAPI for cell nucleus and FITC-conjugated antibody for type II collagen (COL II) in the cartilage part (**B**) of cell-seeded OC scaffolds. The samples were harvested from nude mice 4, 8, and 12 weeks post-implantation. BMSCs and chondrocytes were co-cultured in the OC scaffold and bone and cartilage parts of the scaffolds were observed by confocal microscope. White arrows indicate enhanced OCN deposition into pores. The same staining results were shown for acellular controls in (**C**). Bar = 300 μm.

Figure 12A,B are images of FITC-labelled staining of OCN and COL II at 4, 8, and 12 weeks, with separate images of DAPI staining of cell nucleus. The 4-week samples showed a complete distribution of OCN deposition over the microspheres with concomitant extension to the surrounding pore space. The mono-dispersed DAPI-stained nucleus observed alongside OCN asserts the multi-layered osteoblasts distribution inside the scaffolds. Unchanged spherical morphology of scaffolds further cross-confirms the relatively higher physical integrity of the scaffolds even after 4 weeks implantation, as observed in SEM images of the 4-week bone part in Figure 11B. At week 8, OCN extended more to the surroundings from deformed sphere surface, revealing enhanced OCN deposition into pores (white arrows). This could be due to the degradation of microspheres along with faster differentiation of BMSCs. Nevertheless, the OCN signal of 12-week harvested samples was dramatically higher in comparison with previous time points. Spherical morphology had completely disappeared and thick OCN distribution was found throughout the scaffold, which could also be interrelated with the scaffold morphology observed for the 12-week bone part samples in Figure 11B. A similar trend in COL II deposition was observed for cartilage regeneration (Figure 12B). As in the bone part, the intensity of cell secreted COL II in the cartilage part of OC scaffolds increased with time, reaching the maximum at week 12. The wide dispersal of COL II at this time point coincides with the 12-week OCN deposition in the bone part and affirms the effective regeneration of both tissues simultaneously. The cell-seeded scaffolds were compared with acellular controls in Figure 12C to eliminate the false positive staining of bone and cartilage marker proteins. DAPI nuclear stains were observed due to the host cell infiltration to the interior of the scaffold, however, there was no positive OCN and COL II staining observed for the control, confirming the effective regeneration of osteochondral tissue regeneration in vivo.

#### 3.4.3. Histological Analysis

**Figure 13:**
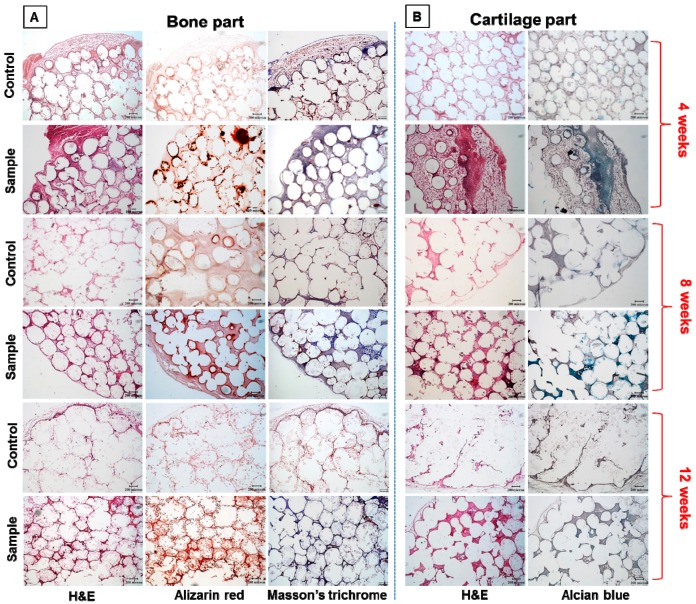
In vivo histological staining of bone (**A**) and cartilage (**B**) parts of co-cultured BMSCs and chondrocytes in OC scaffolds. H&E, Alizarin red, and Masson’s trichrome stains of the bone part and H&E and Alcian blue stains of the cartilage part are shown for the cell-seeded sample and the acellular control 4, 8, and 12 weeks post-implantation in nude mice. Bar = 200 μm.

The osteochondral regeneration was finally confirmed through the histological staining of bone and cartilage parts of OC scaffolds through H&E, AR, AB, and Masson’s trichrome staining. Figure 13A,B respectively represents the histological staining images of the BMSCs-seeded bone part and chondrocytes-seeded cartilage part of the OC scaffolds, harvested 4, 8, and 12 weeks post-implantation. H&E, AR, and Masson’s trichrome were used for the bone part while H&E and AB were selected for the cartilage part. All staining images of samples were compared with acellular controls. Considering the H&E staining, both bone and cartilage parts revealed significant cell growth inside the scaffold at all implantation durations. H&E is the prime clue for osteoid formation with osteoblasts in the bone matrix, which can be very well observed as the purple background at 4, 8, and 12 weeks. It confirmed cell migration and proliferation, with drastic change in cell number in comparison with acellular controls. The purple color observed at 4 weeks in both bone and cartilage parts is the surrounding tissue entrapped in the tissue slice, which can be confirmed through the absence of microspheres around that area. The intensity of H&E stain at week 8 and 12 of samples for bone and cartilage parts was significantly higher than for controls as well as for the 4-week samples. However, deformed sphere shapes observed at both parts of the scaffolds confirms the biodegradation of the scaffold at 12 weeks, as observed from SEM images in Figure 11B. The blurring of the purple color intensity toward the center of degraded microspheres at 12 weeks displays the uniformly wide dispersion of regenerated tissues in both regions.

This trend was followed in AR and Masson’s trichrome stains for bone and AB stain for cartilage. Positive AR staining can reassure the capability of OC scaffolds to continuously support the differentiation of BMSCs into the osteogenic lineage, by displaying bright red spots on the tissue matrix. Basically, AR stain can attach Ca^2+^ ions present in the mineralized bone part, which stems from the differentiation of BMSCs to osteoblasts. Indeed, the mineralized bone part was positively stained with ARS dye and exhibited a linear dependence of stain intensity with duration of implantation. Similar to H&E staining, AR dye had a larger area of staining at week 8 and showed the maximum at a later stage of week 12, endorsing the time-dependent mineralization of BMSCs in bone parts of OC scaffolds. Together with the negative AR staining results of controls at any time point post-implantation, we affirm bone regeneration in the bone part of the co-cultured OC scaffolds in vivo. Masson’s trichrome staining was used to detect the presence of collagen in regenerated bone tissue. It could reveal the presence of osteoids as blue stains in the sections of cell-seeded OC scaffolds when BMSCs are differentiated into osteoblasts. The intensity of blue color at the sphere interfaces was much inferior at 4 weeks, but intensified at 8 and 12 weeks. At week 8, the collagen deposition was higher in larger scaffold pores but fewer where sphere–sphere bonding density was lower. At the later stages of implantation, at 12 weeks, the entire sphere interfaces were uniformly stained with Masson’s trichrome, with much higher intensity than week 4 and 8, which reveals the potential of co-cultured OC scaffolds to regenerate bone in this region.

AB stain highlights the chondrocyte excreted GAGs in the cartilage part of implanted scaffolds (Figure 13B). The AB blue staining intensity of the sample had a slight increment compared to the control at week 4. However, both the control and sample had stable microsphere morphology as observed for the bone part at the same time point. After 8 weeks, degradation of spheres and restoration of cartilage was observed in the sample, compared to the control. The GAGs are expected to be secreted from the newly formed chondrocytes. Though the density of lacuna was lesser, the available staining area at the microsphere interface was considerably sufficient to emphasis the presence of uniformly regenerated cartilage tissue in the OC scaffolds. However, reduction in intensity of the AB-stained cartilage part at week 12 might be due to the larger number of empty spaces created by partially degraded microspheres in the scaffolds. The possibility of a deceleration in cartilage formation at week 12 was ruled out by the existence of high density COL II deposition found at the same time, from IF staining of COL II in Figure 12B. Thus, the histology results specify the efficacy of co-cultured OC scaffolds towards osteochondral tissue formation, which is conferred through the synergistic effect of pre-differentiated BMSCs in the bone part and chondrocytes in the cartilage part. Using PLGA microsphere-based scaffolds containing a continuous gradient in both material composition (nHAP) and encapsulated growth factors, an in vivo study showed that scaffolds with gradients in both material composition and bioactive signals led to faster osteochondral regeneration, and resulted in restoration of the overlying cartilage with quality integration between the cartilage and bone [61].

## 4. Conclusions

This osteochondral tissue engineering is an exciting research area that develops functional strategies for the osteochondral-related clinical issues. In this study, PLGA-based osteochondral scaffolds have been thoroughly investigated for its osteochondral re-modelling efficiency in vitro and in vivo. Three types of scaffolds, i.e., V from PLGA microspheres, C from PLGA/nHAP microspheres, and OC with a combination of PLGA and PLGA/nHAP microspheres, were fabricated through heat sintering using microspheres of size 250–500 µm. TEM observations confirmed the uniform morphology of nHAP, while SEM revealed the smooth and rough morphology of PLGA and PLGA/nHAP microspheres. Both FTIR and XRD data ensured the presence of nHAP in C and OC scaffolds, while elemental analysis confirmed ideal calcium–phosphate ratio of entrapped nHAP. Loading of nHAP in PLGA/nHAP microspheres was estimated to be ~10% by TGA, whereas elemental mapping portrayed the pre-designed arrangement of PLGA/nHAP and PLGA microspheres in OC scaffolds. Mechanical testing of the scaffolds displayed maximum strength for V and minimum for C while OC scaffolds had the value in between both. Live/Dead cell assays of V and C scaffolds single-cultured with chondrocytes, and BMSCs showed similar cell attachment and viability, irrespective of scaffold type. Cell proliferation studies using BMSCs in OM pointed toward the possible mineralization of BMSCs after two weeks of culture and thereby validated the BMSCs culture duration in the bone part of OC scaffolds, prior to chondrocyte seeding. Morphology of pre-differentiated BMSCs in the bone part of OC scaffolds was found unaltered even after co-culture, and based on these results, calcium deposition in the mineralized bone part and GAGs-deposited cartilage part was cross-confirmed through AR staining/CPC treatment and AB staining, respectively. Co-cultured cells in OC scaffolds were found to be viable through Live/Dead assays, while cells with round morphology were observed from cytoskeletal F-actin staining. The presence of COL I/OCN in the bone part and COL II in the cartilage part through IF staining showed the potential of OC scaffolds for biphasic tissue development through co-culture. Most importantly, the positive IF staining and histology results of the newly-formed tissues from the explanted constructs from nude mice endorsed the advantage of hierarchically designed microsphere-based biphasic scaffolds towards effective regeneration of osteochondral tissue.

We concluded that microsphere-based bi-phasic tissue development has a high potential in osteochondral tissue repair. Further studies with graded nHAP concentration and barriers at the bone–cartilage interface zones are promising towards effective remodeling of osteochondral defects in a clinical scenario. Further modifications of the scaffold design with an osteo-conductive gradient in a mechanically tuned bone part and hydrogel-based cartilage part may even have more impacts in the regeneration potential of complex osteochondral defects in vivo.
